# Resistance and Endurance Exercise Training Induce Differential Changes in Gut Microbiota Composition in Murine Models

**DOI:** 10.3389/fphys.2021.748854

**Published:** 2021-12-24

**Authors:** Javier Fernández, Manuel Fernández-Sanjurjo, Eduardo Iglesias-Gutiérrez, Pablo Martínez-Camblor, Claudio J. Villar, Cristina Tomás-Zapico, Benjamin Fernández-García, Felipe Lombó

**Affiliations:** ^1^Department of Functional Biology, Microbiology, University of Oviedo, Oviedo, Spain; ^2^Health Research Institute of the Principality of Asturias (ISPA), Oviedo, Spain; ^3^Instituto Universitario de Oncología del Principado de Asturias (IUOPA), University of Oviedo, Oviedo, Spain; ^4^Department of Functional Biology, Physiology, University of Oviedo, Oviedo, Spain; ^5^Department of Biomedical Data Science, Geisel School of Medicine at Dartmouth, Hanover, NH, United States; ^6^Department of Morphology and Cell Biology, Anatomy, University of Oviedo, Oviedo, Spain

**Keywords:** resistance exercise, endurance exercise, murine models, metagenomics, physical performance

## Abstract

**Background:** The effect of resistance training on gut microbiota composition has not been explored, despite the evidence about endurance exercise. The aim of this study was to compare the effect of resistance and endurance training on gut microbiota composition in mice.

**Methods:** Cecal samples were collected from 26 C57BL/6N mice, divided into three groups: sedentary (CTL), endurance training on a treadmill (END), and resistance training on a vertical ladder (RES). After 2 weeks of adaption, mice were trained for 4 weeks, 5 days/week. Maximal endurance and resistance capacity test were performed before and after training. Genomic DNA was extracted and 16S Ribosomal RNA sequenced for metagenomics analysis. The percentages for each phylum, class, order, family, or genus/species were obtained using an open-source bioinformatics pipeline.

**Results:** END showed higher diversity and evenness. Significant differences among groups in microbiota composition were only observed at genera and species level. END showed a significantly higher relative abundance of *Desulfovibrio* and *Desulfovibrio* sp., while *Clostridium* and *C. cocleatum* where higher for RES. Trained mice showed significantly lower relative abundance of *Ruminococcus gnavus* and higher of the genus *Parabacteroides* compared to CTL. We explored the relationship between relative taxa abundance and maximal endurance and resistance capacities after the training period. *Lachnospiraceae* and *Lactobacillaceae* families were negatively associated with endurance performance, while several taxa, including *Prevotellaceae* family, *Prevotella* genus, and *Akkermansia muciniphila*, were positively correlated. About resistance performance, *Desulfovibrio* sp. was negatively correlated, while *Alistipes* showed a positive correlation.

**Conclusion:** Resistance and endurance training differentially modify gut microbiota composition in mice, under a high-controlled environment. Interestingly, taxa associated with anti- and proinflammatory responses presented the same pattern after both models of exercise. Furthermore, the abundance of several taxa was differently related to maximal endurance or resistance performance, most of them did not respond to training.

## Introduction

Regular exercise is strongly associated with a lower risk of mortality (Myers et al., 2002), in addition to a reduced incidence of most prevalent chronic pathologies in developed countries (Fiuza-Luces et al., 2013; Pedersen and Saltin, 2015). On the other hand, gut microbiota alterations have emerged as one of the common drivers in several highly prevalent conditions, such as metabolic and cardiovascular diseases, as well as in ageing (Qin et al., 2012; O’toole and Jeffery, 2015; Tang et al., 2019). Thus, exercise and gut microbiota have common points of incidence in pathology and health. However, the exact mechanisms through which they carry out this protective effect are not yet entirely elucidated.

In humans, it has been described that regular exercise, mainly endurance (aerobic) exercise, modifies both the general diversity and the abundance of certain gut bacterial phyla or families (Barton et al., 2018). These changes are independent of diet, but they can be reversed once the exercise regime is ceased (Allen et al., 2018). Moreover, acute changes in gut microbiota composition have been also described after a highly exigent endurance exercise such as marathon, precisely in relation to the acute changes in the metabolic demand of this activity (Zhao et al., 2018; Scheiman et al., 2019), suggesting that exercise induces gut microbiome adaptations in a relatively fast way. However, the difficulty in isolating the effect of exercise on human gut microbiome, due to the presence of confounding environmental factors, such as diet (Flint et al., 2015), alcohol and drug use, and even anthropometric characteristics (Rothschild et al., 2018), has led researchers to consider the use of animal models, mainly murine. Although obvious interspecific differences are present, using mouse models for a better comprehension of exercise-induced gut microbiome changes shows many advantages, as reviewed by Nguyen et al. (2015).

Nevertheless, the extrapolation of mice gut microbiota data to humans must be developed with caution. In fact, we have recently highlighted the relevance of having a good knowledge of the composition of the microbiota in mice before performing exercise studies (Fernandez-Sanjurjo et al., 2020), since differences in gut microbiota composition between humans and mice may lead to biased results (Hildebrand et al., 2013; Nguyen et al., 2015; Scheiman et al., 2019). In this regard, several studies have been done exploring the effect of endurance exercise in the mouse gut microbiota under different genetic backgrounds and substrains (Hildebrand et al., 2013). However, these works suffer also from confounding factors that make it difficult to compare between different mouse studies. Thus, differences between endurance exercise protocols, such as voluntary wheel running (VWR) and forced treadmill running (FTR), intensity and duration within sessions, time of intervention, diet composition, and even genetic background (Hildebrand et al., 2013), give rise to different results along the mouse studies on the effect of this model of exercise on gut microbiota profile (Choi et al., 2013; Evans et al., 2014; Allen et al., 2015; Campbell et al., 2016; Lamoureux et al., 2017). Therefore, only when VWR and FTR are compared within the same study, and all other variables are controlled, it can be considered that differences due to distinct endurance exercise regimes in mouse gut microbiota are really observed (Allen et al., 2015; Lamoureux et al., 2017).

Given this consideration regarding mouse models of endurance exercise, where the most common approaches differently affect gut microbiota, there is still a lack of information regarding resistance exercise. Despite its overwhelming relevance for health (Westcott, 2012), the effect of regular resistance exercise on gut microbiota composition has not been analyzed in experimental animal models, although some authors have explored this question in humans, with divergent results (Bycura et al., 2021; McKenna et al., 2021).

Considering the markedly different physiological, metabolic, and molecular mechanisms involved in the response to resistance in contrast to endurance training (Hawley et al., 2014), we hypothesize that both training modalities will exert differential changes in gut microbiota composition. Therefore, the aim of this study was to explore and compare the effect of long-term resistance and endurance training on gut microbiota composition in mice, under controlled conditions, such as diet composition and genetic background.

## Materials and Methods

### Animals and Experimental Design

A total of 26 C57BL6N male mice, 8-week-old, were randomly divided into three groups: sedentary control (CTL, *n* = 6), resistance training (RES, *n* = 8), and endurance training (END, *n* = 12).

Mice were maintained on a 12 h light/dark cycle (onset at 8:00 AM) and under controlled temperature (22 ± 2°C) at the Animal Facilities of the University of Oviedo, Spain (authorized facility No. ES330440003591). All procedures were conducted during the early light portion of the cycle and performed in accordance with the institutional guidelines approved by The Research Ethics Committee of the University of Oviedo, Spain (PROAE 10/2016). Mice were fed a pellet rodent diet (Teklad Irradiated Global 18% Protein Rodent Diet, Envigo, Spain) and water *ad libitum*. The food intake and body weight were measured weekly.

### Training Devices

Endurance training was performed on a treadmill without any aversive stimuli. We used a four-lane commercial rat treadmill (TSE Systems, Germany), with adjustable speed and slope. Rat treadmill is adequate for running four mice at the same time in a lane, maintaining the cage’s group. Resistance training was carried out in an own-manufactured ladder, as described by Codina-Martinez et al. (2020).

### Training Protocols

The same researcher handled and trained the mice during the different stages of training, as previously described by Codina-Martinez et al. (2020): acclimation period, physical performance tests (pre- and post-training), and training protocols.

#### Acclimation Period

Before starting the training program, mice were acclimatized to the training devices for 2 weeks, 5 sessions per week, and 15 min per training session (Kregel et al., 2006). This period was designed to maintain training load to a minimum, avoiding training adaptations that could interfere with pre-training maximal performance tests and guaranteeing no mouse rejects forced exercise training (Kregel et al., 2006).

During the first week, mice were placed on the treadmill without movement and in the resting area at the top of the ladder for periods of 20 min. The following week, acclimatation with movement was performed. Thus, mice walked on the treadmill at 6 m/min. Additionally, they were taught to climb the ladder from the 5th top step to the resting area, increasing the number of climbed rungs gradually to 10. A piece of clinical tape was placed on their tails, where the weight will be placed during the training period, as they climbed the ladder to familiarize the mice with this procedure. After a few days, a lightweight load (5 g) was attached to the mice’ tails with clinical tape. Mice were adapted to run on the treadmill and to climb the ladder without any aversive stimuli throughout all stages of the training protocol, to diminish confounder factors and favoring repeatability maximal tests (Kregel et al., 2006; Knab et al., 2009; Conner et al., 2014; Allen et al., 2015), allowing us to train all the mice without refusals.

#### Maximal Performance Tests

Forty-eight hours after the end of the acclimation period, mice were randomly distributed in the above-mentioned groups. Then, those at the END and RES groups performed a maximal endurance or resistance test, respectively.

Maximal endurance capacity was determined by an incremental test in the treadmill, as described before by Codina-Martinez et al. (2020). After a 10-min warm-up at 9 m/min with 10° slope, the incremental test started at 12 m/min. Every 3 min speed was increased by 3 m/min, until exhaustion. Maximum speed (m/min) and total time (min) were recorded, to calculate the total distance (m) as a measurement of endurance capacity.

Maximal resistance capacity was tested in the ladder, following a protocol adapted from previous studies (Codina-Martinez et al., 2020). Mice performed a warm-up consisting of 3 series of 10 repetitions, 10 steps/repetition, at 90° of slope, without external load. The mice rested for 60 s between series. Then, the slope was set at 85° and the mice performed successive series of 10 steps with increasing external loads until exhaustion. The starting external load was 10 g, increasing 5 g in each series. The mice rested for 120 s in the resting area after each series. If the mice failed to climb 10 steps with a particular weight load, they were allowed another try with the same load after 120 s of rest. If they failed again, the weight load of the last complete series was recorded as their maximal weight load. The maximal resistance capacity was expressed as the maximal weight load relative to body weight (%).

Both tests were repeated at the end of the training period, following the same protocols.

#### Training Protocols

All END and RES mice were trained for 4 weeks, 5 days/week (Monday to Friday). Training protocols were adapted from previous works (Kregel et al., 2006; Codina-Martinez et al., 2020) in terms of intensity and duration of sessions. To reduce mice anxiety, they were trained in groups of four animals from the same cage. Aversive stimuli were also avoided, to minimize stress.

Endurance training sessions started with identical warm-up as for the maximal endurance performance test. Then, all sessions of continuous running had a mean duration of 60 min and the distance covered every day was 1,000 m. However, the intensity in terms of maximal speed, number of stages, as well as the speed and duration of each stage, varied along the week according to this structure: 2 days at high intensity (Tuesday and Friday), 2 days at moderate intensity (Monday and Thursday), and 1 day at low intensity (Wednesday). Speed ranged from 12 to 24 m/min, which corresponded to 40–80% of mean maximal speed at the pre-training test (Kemi et al., 2002). The duration of each stage varied inversely with speed, between 15 and 5 min (Kemi et al., 2002). The slope was fixed at 10°. Maximal intensity increased throughout the training period, although maintaining the weekly schedule and the duration and the distance covered in training sessions.

Resistance training sessions started with an identical warm-up as for the maximal resistance performance test. Then, all sessions were designed to achieve the same exercise volume through a combination of the number of steps climbed (or distance against gravity) and weight load (Figueiredo et al., 2018). Considering the combination of these parameters, an accumulated work of 260 mJ (g m^2^/s^2^), was achieved daily. The number of steps per training session varied between 400 and 2,000 depending on the maximal weight load, which ranged between 20 and 50 g or 25–65% of the maximal weight load at the pre-training test. We selected these maximum weight ranges because it has been described that below 75% of 1 repetition maximum there is no velocity loss, which is important for standardizing intensity of submaximal efforts (Gentil et al., 2018). Week planning was: 2 days with high weight load and low number of steps (Tuesday and Friday), 2 days of intermediate weight load and the number of steps (Monday and Thursday), and 1 day without weight load but a high number of steps (Wednesday). The number of steps and the maximum weight loads increased throughout the training period, although maintaining the weekly schedule, as well as the accumulated work and the percentages of maximum weight load.

CTL mice remained in a cage, in the same room, while END and RES groups were training.

### Genomic DNA Extraction and 16S rRNA Sequencing for Metagenomics

All mice were sacrificed at 16 weeks of age by CO_2_ inhalation, 24 h after the last exercise bout. Caeca were extracted and preserved at −80°C until extraction. E.Z.N.A.® DNA Stool Kit (VWR International, Spain) was used for genomic DNA (gDNA) extraction (200 mg of frozen cecal content). A BioPhotometer® (Eppendorf Ibérica S.L.U., Spain) was used for gDNA quantification, a prior step before preparing working solutions diluted to 6 ng/μl, which were needed for PCR amplification. 16S rRNA PCR amplification was carried out with the 16TM Metagenomics Kit (A26216, Fisher Scientific, Spain), using 7 out of 9 hypervariable regions in this gene, *via* two PCR amplifications (two sets of oligonucleotides pools: on one side, the regions V2, V4, and V8 (amplicons 250, 288, and 295 bp respectively); and on the other side the regions V3, V6-V7, and V9 (amplicons 215, 260, and 209 bp respectively). PCR amplicons were used to generate a library (Ion Plus Fragment Library kit for AB Library Builder™ System, Fisher Scientific). The indexing of each sample was carried out with the Ion Xpress™ Barcode Adapters 1–96 kit (Thermo Fischer Scientific). The ION OneTouch™ 2 System and the ION PGM™ Hi-Q™ OT2 kit (Fisher Scientific) was used for preparing the templates. The ION™ PGM Hi-Q™ Sequencing kit (Fisher Scientific) on the ION PGM™ System was used for metagenomics sequencing. The ION 318™ v2 Chip (Fisher Scientific) was used (Fernandez et al., 2018).

For each mouse metagenomics, the consensus spreadsheet (ION Reporter software 5.6, Fisher Scientific, Spain) included the percentages for each phylum, class, order, family, or genus/species. Shannon and Simpson diversity indexes were calculated using ION Reporter software. These data were used to compare frequencies between groups. Taxonomic adscription up to species level was performed using the QIIME 2 (v.2017.6.0) open-source bioinformatics pipeline (Caporaso et al., 2010). The reference library used was the Curated MicroSEQ(R) 16S Reference Library v2013.1; Curated Greengenes v13.5 (Fernandez et al., 2018). The number of mapped reads (after the ignored ones due to less than 10 copies) per sample was always over 100,000. Counts were normalized by sum scaling. All raw metagenomics data have been deposited at NCBI SRA database (accession number PRJNA558220).

For further analysis, only those taxa with a relative abundance ≥0.1% in at least one of the groups were used (Supplementary Table S1).

### Statistical Analysis

Normality of the variables was tested by means of the Shapiro–Wilk test. Initial comparison between the three experimental groups for families, species and genera were performed using PERMANOVA tests. Principal component analysis was used for dimension reduction. Bivariate representation of the participant using the two main components are also provided. Comparison between the three experimental groups was performed using one-way ANOVA test with Tukey’s *post hoc* test. Spearman’s correlation coefficient was calculated to explore associations between variables. Graph Pad Prism 8 (Graph Pad Software, La Jolla, CA, United States) and R.4.01 (www.r-project.org) including the PERMANOVA package were used for statistical analysis. Differences between groups were considered significant when value of *p* < 0.05. The statistical significances are indicated in the text and in the figures.

## Results

### Trained Mice Characteristics

**Figure 1:**
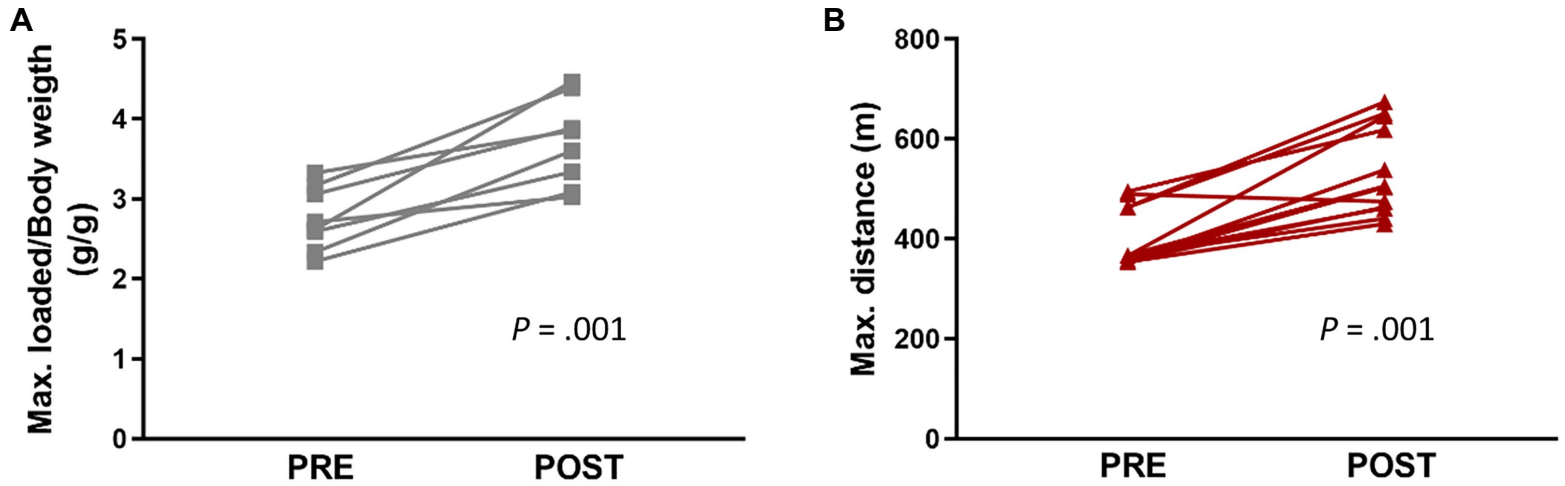
Maximal performance tests developed before (Pre) and after (Post) exercise intervention in resistance (**A**, *n* = 8) and endurance (**B**, *n* = 12) groups. Each dot represents one mouse. Statistically significant differences are marked with the value of *p* for each comparison.

During the four-week training period, no differences were observed among the three groups (CTL, END, and RES) in daily food intake or body weight. All groups increased their body weight at the end of the intervention with respect to baseline (data not shown). Regarding physical capacities, both END and RES groups significantly improved the respective trained capacity after the four-week training period (Figure 1).

### An Overview of Gut Microbiome Composition

Considering all mice data, cecal 16S metagenomic dataset yielded a total of 11 phyla, 21 classes, 42 orders, 90 families, 64 genera, and 68 species. For further analysis, only those taxa with a relative abundance ≥0.1% were used (Supplementary Table S1).

**Figure 2:**
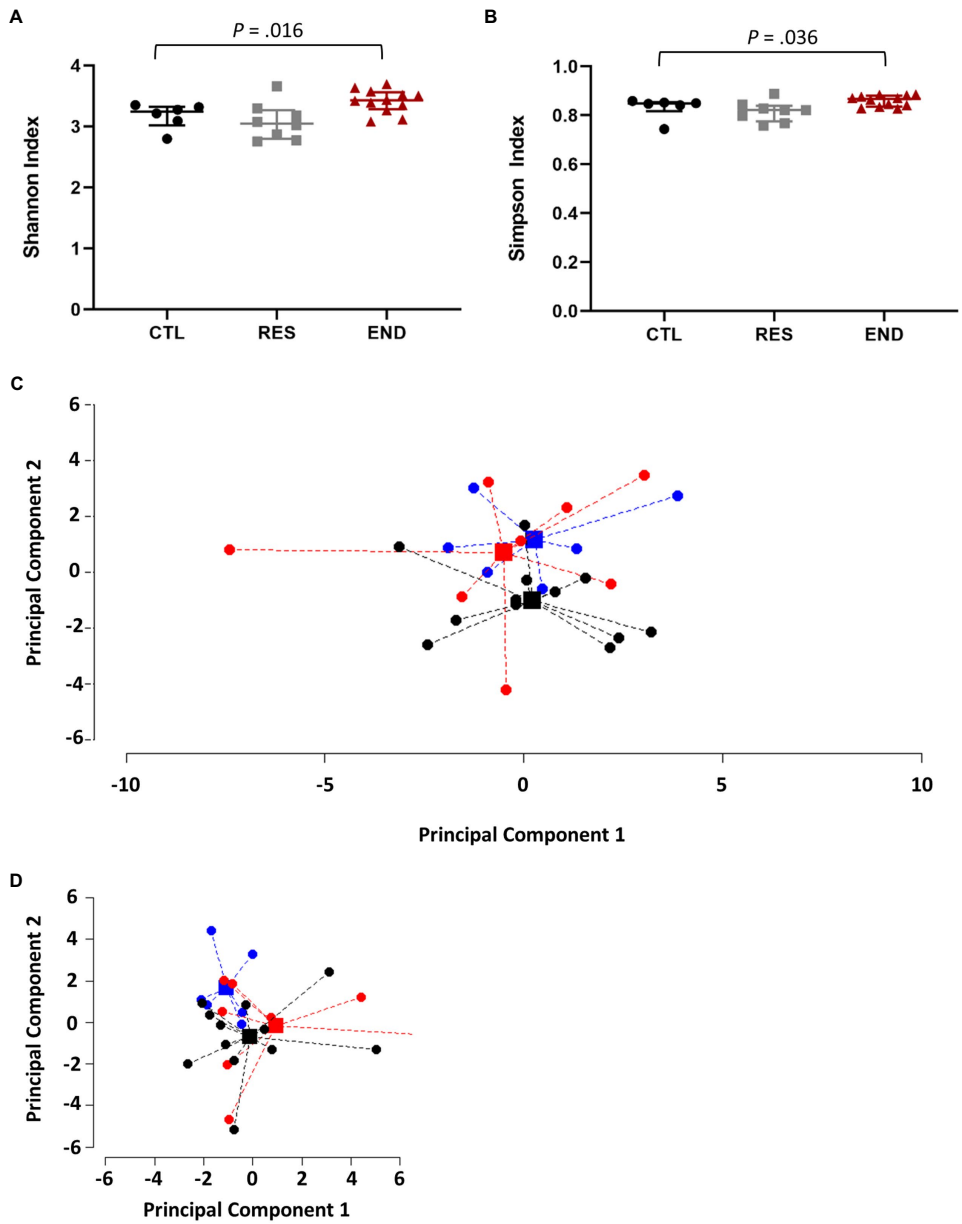
Gut microbiome composition. **(A)** Gut microbiome diversity using the Shannon index. **(B)** Gut microbiome evenness using the Simpson index. **(C)** Points and centroids of the PERMANOVA analysis for the three different groups projected on the two main dimensions of the principal component analysis (PCA) at family level. **(D)** Points and centroids of the PERMANOVA analysis for the three different groups projected on the two main dimensions of the principal component analysis (PCA) at genera and species levels. CTL: control (*n* = 6), RES: resistance (*n* = 8), END: endurance (*n* = 12). Data are presented as mean ± SEM. Each dot represents one mouse. Statistically significant differences are marked with the value of *p* for each comparison.

Gut microbiota biodiversity was determined by Shannon and Simpson indexes. Our results show that END mice presented significantly higher values for both indexes in comparison to CTL mice (Figures 2A,B). Although Shannon and Simpson indexes reflect diversity, Shannon index has a higher sensitivity to species richness, whereas Simpson index to species evenness (Johnson and Burnet, 2016), suggesting that not only END mice had more different species within their microbiome, but also that this diversity is more uniform. No differences among training groups were found at phylum, class, order, nor family level. For instance, at family level, the PERMANOVA gives a value of *p* of 0.086. Figure 2C shows the points and the centroids for the three different groups projected on the two main dimensions of the principal component analysis (PCA) for the families, which explain a 30% of the total variance. *Firmicutes* (~60%) and *Bacteroidetes* (~30%) were the most abundant phyla in all mice, followed by *Proteobacteria* (~5%), whereas *Lachnospiraceae* (~24%), *Phorphyromonaceae* (~16%), *Clostridiaceae* (~14%), *Lactobacillaceae* (~9%), and *Bacteroidaceae* (~8%) account for the most abundant families. Significant differences among training groups were observed at genus and species level (value of *p* of 0.009). In Figure 2D, genera and species in the first two main dimensions of PCA, which explain 37% of the total variance, are plotted.

### Comparing Exercise Models

**Figure 3:**
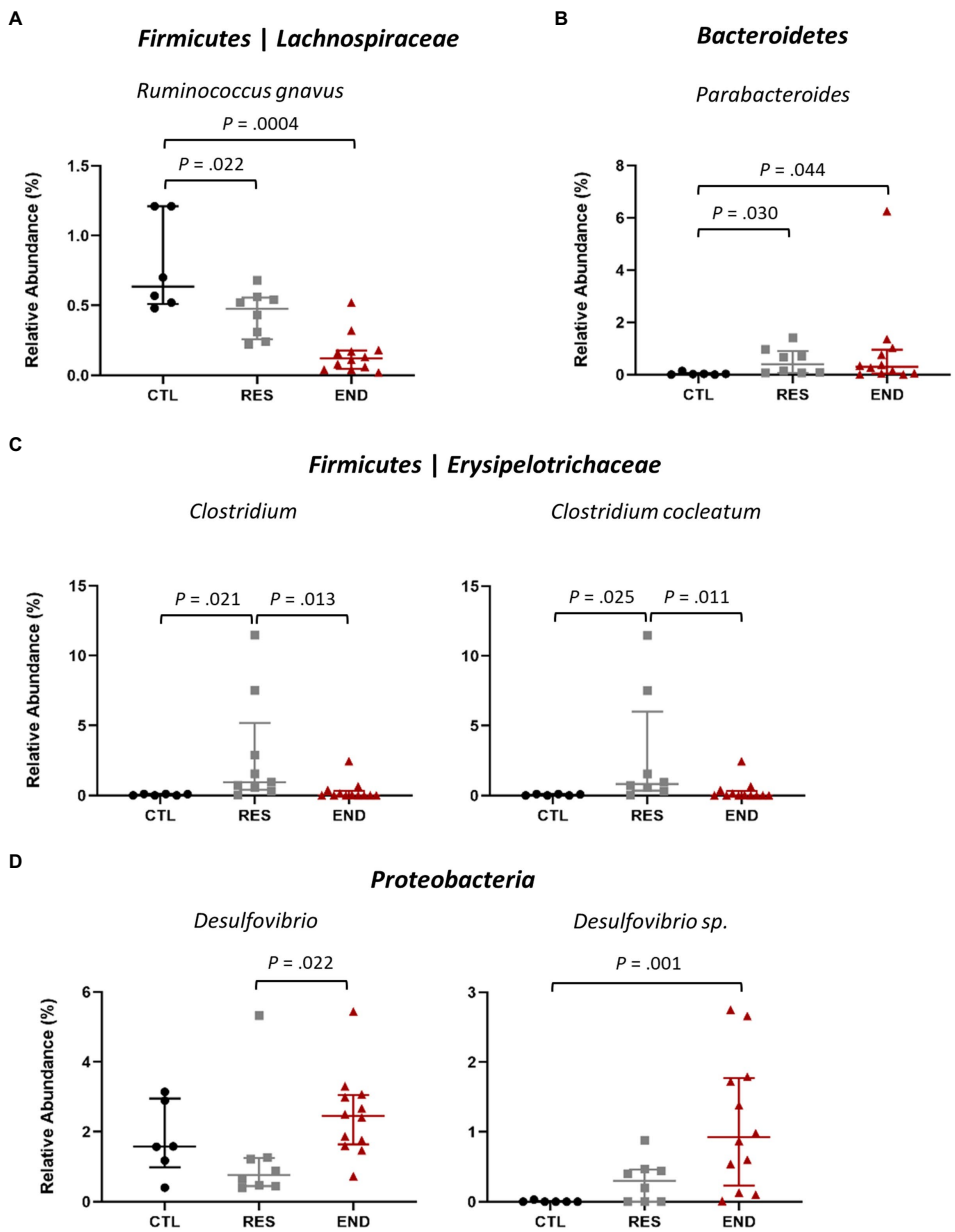
Relative abundance at genus and species after exercise intervention. **(A)** *Ruminococcus gnavus*. **(B)** *Parabacteroides*. **(C)** *Clostridium* and the species *C. cocleatum*. **(D)** *Desulfovibrio*, including the taxon *Desulfovibrio* sp. CTL: control (*n* = 6), RES: resistance (*n* = 8), END: endurance (*n* = 12). Data are presented as mean ± SEM. Each dot represents one mouse. Statistically significant differences are marked with the value of *p* for each comparison.

Within *Firmicutes* phylum, both END and RES mice showed significantly less relative abundance of the species *Ruminococcus gnavus* regarding CTL (Figure 3A). Interestingly, within the second most abundant phylum, *Bacteroidetes*, the genus *Parabacteroides* presented higher relative abundance in both END and RES groups as compared to CTL (Figure 3B).

RES group displayed higher relative abundance of *Clostridium* and *C. cocleatum* compared to CTL and END (Figure 3C), while *Desulfovibrio* and *Desulfovibrio* sp., belonging to the phylum *Proteobacteria*, yielded higher relative abundance in END group regarding RES and CTL (Figure 3D).

### Correlations Between Gut Microbiome Relative Abundance and Physical Performance

**Figure 4:**
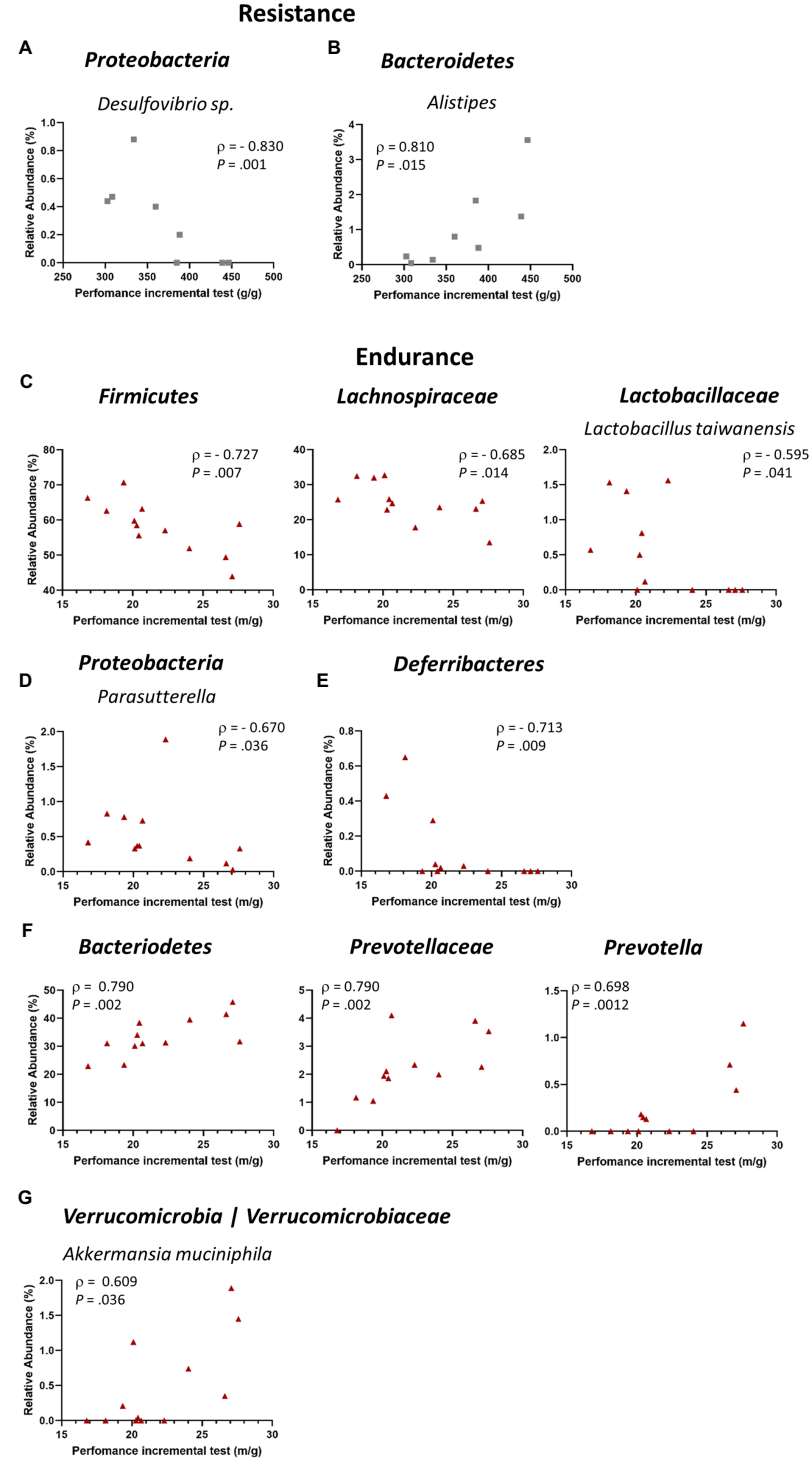
Correlations between gut microbiome relative abundance and physical performance. **(A,B)** Correlations between relative abundance and final resistance performance (*n* = 8); **(A)** *Proteobacteria* (species *Desulfovibrio* sp.); **(B)** *Bacteroidetes* (genus *Alistipes*). **(C–G)** Correlations between relative abundance and final endurance performance (*n* = 12); **(C)** *Firmicutes* (including the family *Lachnospiraceae* and the species *L. taiwanensis*); **(D)** *Proteobacteria* (genus *Parasutterella*); **(E)** *Deferribacteres*; **(F)** *Bacteroidetes* (including the family *Prevotellaceae* and the genus *Prevotella*). Each dot represents one mouse. Spearman’s rank correlation coefficient, ρ, and value of *p* are shown for each correlation.

We further explored the possible correlation of the different detected taxa with performance in the incremental test after exercise intervention. Thus, we estimated the relationship between the cecal microbiota relative abundance detected after exercise training and the physical performance of the mice at the post-training test, corrected by the body weight of each mouse. When considering RES group, we found that the *Proteobacteria* taxon *Desulfovibrio* sp. had a negative correlation with resistance performance (Figure 4A), while the genus *Alistipes* (phylum *Bacteroidetes*) was positively correlated (Figure 4B). Interestingly, END mice presented a higher number of taxa which correlated with physical performance (Figure 4C). For instance, *Firmicutes* phylum showed a negative correlation with endurance performance, and such negative correlation was also seen in the family *Lachnospiraceae* and in the species *Lactobacillus taiwanensis*, from the *Lactobacilljaceae* family. Additionally, the genera *Parasutterella*, a *Proteobacteria*, and the phylum *Deferribacteres* also presented a negative correlation with endurance final performance test (Figures 4D,E). On the other hand, the phylum *Bacteroidetes* showed a strong positive correlation with endurance performance, being the family *Prevotellaceae* and the genus *Prevotella* great contributors to this correlation (Figure 4F). Lastly, the phylum *Verrucomicrobia* also displayed a positive correlation with endurance performance, having as its only representative the bacterium *Akkermansia muciniphila* (Figure 4G).

**Figure 5:**
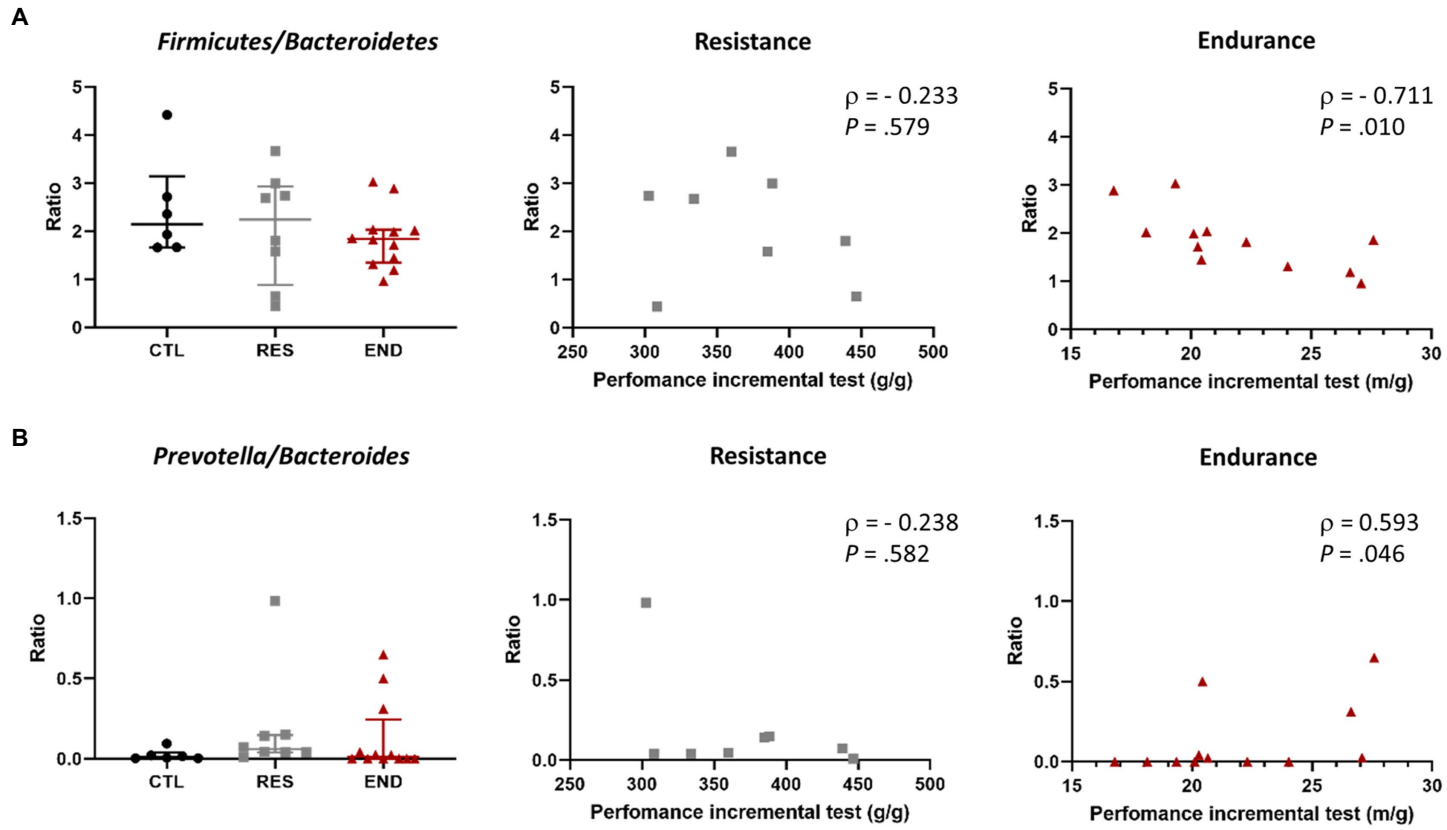
**(A)** *Firmicutes*/*Bacteroidetes* ratio in the three groups, and its correlation with exercise performance in resistance and endurance groups. **(B)** *Prevotella*/*Bacteroidetes* ratio in the three groups, and its correlation with exercise performance in resistance and endurance groups. CTL: control (*n* = 6), RES: resistance (*n* = 8), END: endurance (*n* = 12). Ratios are presented as mean ± SEM. Each dot represents one mouse. Spearman’s rank correlation coefficient, ρ, and value of *p* are shown for each correlation.

The *Firmicutes* to *Bacteroidetes* ratio and the *Prevotella* to *Bacteroides* ratio were calculated. We did not observe differences among intervention groups (Figures 5A,B). Strikingly, maximal endurance capacity at the end of the training period showed a negative correlation with *Firmicutes* to *Bacteroidetes* ratio (Figure 5A), while a positive correlation was observed with the *Prevotella* to *Bacteroides* ratio (Figure 5B).

## Discussion

Murine models used in early microbiome studies in the context of exercise suggested the profound effect intestinal microbiota composition has on exercise performance, since germ-free and gnotobiotic mice showed a lower endurance capacity (Hsu et al., 2015). Similarly, it is known that endurance training induces in gut microbiota composition, both in human (Allen et al., 2018) and mouse (Campbell et al., 2016), while no information is available about resistance exercise in mice. In this regard, Chen et al. (2021) reported that strength training modified the gut microbiota composition of healthy and multiple sclerosis mice, although it seems unlikely that the training device used by the authors allows for specific resistance training. Therefore, these results should be taken with caution regarding the effect of resistance training on gut microbiota composition in mice.

One of the main concerns in the analysis of gut microbiota is the confounding effect of both environmental (Rothschild et al., 2018) and genetic (Hildebrand et al., 2013) variables, that make it difficult to isolate the effect of exercise. In this sense, the analysis of samples obtained from experimental animals has several advantages, despite important interspecific differences in gut microbiota composition with humans (Ley et al., 2005). On the one hand, there is a more feasible possibility of accessing cecal samples, which gives a closer idea of the established microbiota (Marteau et al., 2001). In addition, the use of a homogeneous diet allows the elimination of the confusing effect of different food components (Flint et al., 2015). Finally, experimental animal models are inbred, providing a more homogenous genetic background (Nguyen et al., 2015), considering the strong influence of host genetics on gut microbiome composition (Goodrich et al., 2014). Thus, taking this into consideration, we explored here, for the first time, the effect of long-term resistance training on gut microbiota in mice. We have found that exercise training modifies gut microbiota composition and that some of these changes show a specific profile depending on the capacity most emphasized during training, resistance or endurance.

In our study, endurance training favored both high diversity and evenness, according to Shannon and Simpson indexes, respectively. However, neither END or RES showed differences with CTL in terms of phyla and families compositions. Differences were observed at genus and species levels, suggesting that the potential rearrangements promoted by exercise affect mainly lower taxonomic levels in our model. In fact, we found that some taxa are modified by training, independently of the model of exercise, while others are specific for endurance or resistance training. In this way, the *Firmicutes* genus *Clostridium*, and the species *C. cocleatum*, is specifically changed after resistance exercise. *C. cocleatum* has been previously implicated in the prevention of *C. difficile* infections in mice (Boureau et al., 1993) and it was found to be increased after metformin treatment of high-fat diet mice, positively correlated with AMPKα1 levels in liver (Lee and Ko, 2014), which suggests its possible association with metabolic improvement (Lee and Ko, 2014). Interestingly, the genus *Clostridium* has been earlier shown to be more abundant in cecal samples from a FTR endurance mouse model regarding sedentary mice, although not reaching species level (Allen et al., 2015). In that study, the FTR training design had a lower intensity than our FTR endurance model, and mice were from a different substrain (C57BL/6J vs. C57BL/6N), which can account for the differences observed between both studies (Hildebrand et al., 2013; Table 1). However, we found that high intensity endurance exercise specifically changes the *Proteobacteria* genus *Desulfovibrio*, and the species *Desulfovibrio* sp. *Desulfovibrio* is a hydrogen sulfide (H_2_S) producer, a compound which has been proposed to act, in conjunction with the SIRT1 pathway, in the prevention of endothelial cells age-associated apoptosis after endurance exercise in mice (Das et al., 2018). Thus, H_2_S production by *Desulfovibrio* may improve endothelial function during endurance training, at least in mice, favoring the appropriate oxygen supply to cells, something that deserves to be studied in more depth.

**Table 1:** Comparison of our study and previous studies regarding gut microbiota in mice that have undergone endurance exercise.

Study	Ours	Allen et al., 2015	Lamoureux et al., 2017 ^*^	Allen et al., 2015	Campbell et al., 2016	Evans et al., 2014	Choi et al., 2013
n	12	10	11	10	9	5–6	6
Substrain	C57BL/6 N	C57BL/6 J	C57BL/6 N	C57BL/6 J	C57BL/6 N	C57BL/6 J	C57BL/6 N
Age (weeks)	8	6	6–10	6	6	5	44
Caged	6–8	Individually	Individually	Individually	Individually	Individually	Individually
Diet (energy) Protein Fat Carbohydrates	3.1 kcal/g 24% 18% 58%	3.0 kcal/g 29% 17% 54%	3.20 kcal/g 26% 14% 60%	3.0 kcal/g 29% 17% 54%	3.82 kcal/g 20% 10% 70%	3.85 kcal/g 20% 10% 70%	n.a.
Exercise	FTR	FTR	FTR	VWR	VWR	VWR	VWR
Intervention time (weeks)	4 (5 days/week)	6 (5 days/week)	6 (5 days/week)	6 (5 days/week)	12 (7 days/week)	12 (7 days/week)	5 (7 days/week)
Distance/session	1,000 m	480 m	600-700-800 m	5,836 ± 132 m/night	17,390 ± 6,890 (Counts)	11714.28 m	11538.06 m
Time/session	60 min	40 min	40 min	n.a.	n.a.	n.a.	680 min
Speed	12–24 m/min	8–12 m/min	15–20 m/min	n.a.	n.a.	n.a.	18.67 m/min
Exercise intensity	High	Low	Moderate	n.a.	n.a.	n.a.	
Group training	Yes	No	No	No	No	No	No
Sample origin	cecal	cecal	fecal	cecal	fecal	fecal	fecal
16S rRNA sequencing	V2-4-8, V3-6, and V7-9 sequencing	V3 and V5 sequencing	V6-V8 sequencing	V3 and V5 sequencing	TRFLP and pyrosequencing	TRF and V4 sequencing	PhyloChip Arrays
Relevant taxa (vs. Sedentary mice)	↓*Ruminoccocus gnavus* ↑*Parabacteroides* ↑*Desulfovibrio*	↑*R. gnavus* ↑*Clostridium* ↑*Butyrivibrio* ↑*Oscillospira* ↑*Coprococcus*	↓*Ruminoccocus* ↓*Lactobacillus* ↓*Clostridiales* ↓*Parabacteroides* ↑*Bacteroides* ↑*Lachnospiraceae*	↑*Coprococcus* ↑*Turicibacter*	↑*Allobaculum* spp. ↑*Ruminoccocus* ↑*Clostridiales* ↑*Akkermansia*	Exercise increased the *Bacteroidetes*:*Firmicutes* ratio in a manner that was proportional to the distance run.	↓*Ruminoccocus*

n.a.: Data not available.

* This study includes both male (*n* = 5) and female (*n* = 6) mice, the rest of the studies were conducted on males.

In addition to these model of exercise dependent taxa, other taxonomic groups responded to physical training independently of the model of exercise. In this sense, the increase in anti-inflammatory *Parabacteroides* and the decrease in pro-inflammatory bacterium *Ruminoccoccus gnavus* showed similar patterns for END and RES, suggesting a role of exercise in the modulation of the balance between pro- and anti-inflammatory microbiota (Le Chatelier et al., 2013). Interestingly, a previous study developed in rats subjected to concurrent training also showed an increase in *Parabacteroides* (Carbajo-Pescador et al., 2019). However, differences in the behavior of *R. gnavus* are seen among previous works regarding endurance exercise (Table 1). In fact, our results showed that *R. gnavus* relative abundance is lower in trained than in sedentary mice. These results contrast with other endurance FTR models, of lower intensity, where this species was found increased after endurance exercise (Allen et al., 2015). Interestingly, the genus *Ruminoccocus* has different behavior among endurance studies, since it could appear either increased (Campbell et al., 2016) or decreased (Choi et al., 2013; Lamoureux et al., 2017), reinforcing the fact that comparison among different studies have to be made with great caution in order to understand what the origin of the discrepancies at this level could be (Table 1).

Apart from the analysis of the effect of physical training on gut microbiota composition, another point of interest is the study of the relationship between gut microbiota composition and physical performance. To better understand which taxa are the most relevant in terms of exercise performance, we correlated the relative abundance with resistance or endurance performance after the training intervention (Fernandez-Sanjurjo et al., 2020). Interestingly, most of the performance-related taxa were not modified by training.

Two taxa were found to be correlated with resistance exercise in our model. Interestingly, *Desulfovibrio* sp. was negatively correlated with resistance performance, a species which relative abundance was higher in the endurance exercise group. Thus, this H_2_S producer could be favoring endurance adaptations, through endothelial function improvement (Das et al., 2018), but strikingly it seems to be disadvantageous for resistance exercise, although the exact mechanism is unknown. On the other hand, *Alistipes*, a genus belonging to the *Bacteroidetes* family *Rikenellaceae* was positively correlated with resistance performance. *Alistipes* is one of the newest genera within the phylum *Bacteroidetes*, and it has been implicated in the fermentation of unprocessed proteins within the gut, producing toxic metabolites (Parker et al., 2020). In humans, *Alistipes* has been shown to participate both in health and disease, and some studies in mice suggested its protective role against colitis, as reviewed by Parker et al. (2020). Considering its relationship with protein metabolism, the possible involvement of *Alistipes* in metabolic adaptations to resistance training deserves further analysis.

In the case of endurance exercise, we have previously shown that *Lactobacillaceae* family was negatively correlated with endurance performance when considering only *Firmicutes* phylum (Fernandez-Sanjurjo et al., 2020). Within the same paradigm, we showed in this study that this phylum was indeed negatively associated with endurance performance, and *Lachnospiraceae* and *Lactobacillaceae* families can account for this effect. Moreover, the *Firmicutes*-to-*Bacteroidetes* ratio was also negatively correlated with endurance performance, reinforcing the association between *Firmicutes* and lower endurance capacity. In fact, higher relative abundance of *Firmicutes* over *Bacteroidetes* has been previously associated to obesity in both mice and humans, as reviewed in Crovesy et al. (2020) and Mohr et al. (2020), and for this reason this ratio is considered a marker of health. Accordingly, our results showed that *Bacteroidetes* were positively correlated with endurance performance, and it is possible that *Prevotellaceae* family and *Prevotella* genus may play a role in this. In fact, the relation between the relative abundance of *Prevotella* and the amount of exercise has been previously observed in cyclists, where the higher abundance of *Prevotella*, the higher time exercising per week (Mohr et al., 2020). Higher abundance of *Prevotella* is one of the three enterotypes described in humans, along with *Bacteroides* and *Ruminoccocus* (Arumugam et al., 2011), associated with diet patterns (Wu et al., 2011). In mice, two enterotypes were suggested, *Bacteroides* and *Ruminoccocus*, being the last one associated with C57BL/6 substrains (Hildebrand et al., 2013). Thus, it could be possible that *Prevotellaceae* and *Prevotella* taxa may be implicated in the adaptive response to endurance exercise in both humans (Mohr et al., 2020) and mice. Moreover, the *Ruminoccocus* enterotype observed in C57BL/6 substrains usually coexists with *Akkermansia* (Arumugam et al., 2011). Interestingly, *Akkermansia muciniphila* relative abundance was positively related with endurance performance in our study. This bacterium has been previously shown to promote health in mice, even increasing their lifespan, and it was observed that centenarian subjects presented higher abundance (Barcena et al., 2019). Thus, in light of our results, *Akkermansia* may be also involved in endurance exercise adaptions in mice.

## Strengths and Limitations

Our study has several strengths. First, the design and implementation of a specific and highly controlled strength training model in experimental animals allows us to obtain information, for the first time, on the effect of this type of exercise on the gut microbiota in mice. On the other hand, the use of experimental animals, with a homogeneous genetic background and diet, makes it possible to isolate the effect of exercise on the composition of the gut microbiota, minimizing the influence of confounding variables. In addition, the analysis of caecal samples gives a more approximate idea of the native microbiota compared to fecal samples. Finally, the sequencing analysis of 16S rRNA also allows for a better resolution. In many studies the V4 hypervariable region of 16S rRNA is used as universal amplification zone, allowing representative data to be given at the genus level. However, the combination of data from the different hypervariable regions, as in our study, gives a higher resolution, distinguishing more deeply the different taxa. Including the V1 to V3 regions allows a better resolution of the families detected and the V3 to V5 regions give a greater specificity in the studied prokaryotic kingdom (Colston and Jackson, 2016; Davidson and Epperson, 2018; Grogan et al., 2019).

Some limitations of the present study should be noted. First, a larger sample of individuals would have been desirable, although similar sample sizes, typically 6–10, have been usually reported in studies following endurance training, as it is now shown in Table 1. Since resistance training is more complex than endurance in terms of design and execution, and requires individual animal manipulation, a smaller number of animals were included in the RES group. The CTL group also initially consisted of 8 animals. During sample extraction and processing, two samples were discarded for technical or quality control reasons. Therefore, samples from 6 animals were finally included in the CTL group. Second, generalization of results is limited, since only male mice were included. Given the limited availability of studies in which the effect of exercise on gut microbiota composition in females has been analyzed and considering the potential translational significance and public health relevance of these results, it would be necessary to extend this study to trained females. Finally, to remark that, since the number of studied categories increases with the specificity of the taxonomy considered, the risk of multiplicity/overfitting increases. This study is observational and following the STROBE guidelines, no multi-testing correction has been considered. Therefore, inferential results should be taken with caution.

## Conclusion

Resistance and endurance training modify the composition of the gut microbiota in mice, under a high-controlled environment. In a pioneering approach, we have observed that some taxonomic groups are differently affected by the model of exercise, which opens the possibility for the definition of resistance and endurance exercise gut microbiome profiles. Interestingly, taxa associated with anti- and proinflammatory responses presented the same pattern after both models of exercise, making it also possible to distinguish between sedentary and trained mice and highlighting its role in the anti-inflammatory adaptive response to regular training.

Interestingly, the abundance of several taxa was differently related to maximal endurance and resistance performance, although most of them did not respond to training. This opens the possibility to explore other ways to modulate those taxa associated with performance, e.g., through diet.

All in all, even though exercise murine models can help in understanding the effect of exercise on gut microbiome, differences between studies (e. g. species, diet, genetic background, exercise intensity, and even 16S rRNA sequencing strategy) makes it complicated to compare the results obtained from the different studies, which nowadays limits the possibility of designing oriented interventions. Therefore, as we have previously suggested (Fernandez-Sanjurjo et al., 2020), there is the need for prior in-depth knowledge of the baseline microbiota response to exercise to better understand the effect of potential subsequent interventions oriented towards the modification of gut microbiota composition for health promotion or for performance purposes.

## Data Availability Statement

The datasets presented in this study can be found in online repositories. The names of the repository/repositories and accession number(s) can be found at: https://www.ncbi.nlm.nih.gov/, PRJNA558220.

## Ethics Statement

The animal study was reviewed and approved by the Research Ethics Committee of the University of Oviedo, Spain (PROAE 10/2016).

## Author Contributions

JF and MF-S performed the experiments, analyzed the data, and wrote the manuscript. CT-Z and PM-C analyzed the data, prepared the figures, and wrote the manuscript. CJV analyzed the data. EI-G, FL, and BF-G designed and supervised the study and wrote the manuscript. All authors have read and approved the final version of the manuscript and agree with the order of the presentation of the authors.

## Funding

This work was supported by Ministerio de Economía y Competitividad under Grant DEP2015-69980-P to BF-G and by Programa de Ayudas a Grupos de Investigación del Principado de Asturias to FL (FC-GRUPIN-IDI/2018/000120).

## Conflict of Interest

The authors declare that the research was conducted in the absence of any commercial or financial relationships that could be construed as a potential conflict of interest.

## Publisher’s Note

All claims expressed in this article are solely those of the authors and do not necessarily represent those of their affiliated organizations, or those of the publisher, the editors and the reviewers. Any product that may be evaluated in this article, or claim that may be made by its manufacturer, is not guaranteed or endorsed by the publisher.
