# Supplementary material for: Resistance and Endurance Exercise Training Induce Differential Changes in Gut Microbiota Composition in Murine Models
Source: Front Physiol. 2021 Dec 24;12:748854. doi: 10.3389/fphys.2021.748854 (PMC8739997; doi:10.3389/fphys.2021.748854)
Supplement: Supplementary file 1 [file Table_1.docx]

| **Study** | **Ours** | **Allen *et al*. 2015** | **Lamourex *et al*. 2017*** | **Allen *et al*. 2015** | **Campbell *et al*. 2016** | **Evans *et al*. 2014** | **Choi *et al*. 2013** |
| --- | --- | --- | --- | --- | --- | --- | --- |
| **n** | **12** | **10** | **11** | **10** | **9** | **5-6** | **6** |
| **Substrain** | C57BL/6N | C57BL/6J | C57BL/6N | C57BL/6J | C57BL/6N | C57BL/6J | C57BL/6N |
| **Age (weeks)** | 8 | 6 | 6-10 | 6 | 6 | 5 | 44 |
| **Caged** | 6-8 | Individually | Individually | Individually | Individually | Individually | Individually |
| **Diet (energy)**  **Protein**  **Fat**  **Carbohydrates** | 3.1 kcal/g  24%  18%  58% | 3.0 kcal/g  29%  17%  54% | 3.20 kcal/g  26%  14%  60% | 3.0 kcal/g  29%  17%  54% | 3.82 kcal/g  20%  10%  70% | 3.85 kcal/g  20%  10%  70% | n.a. |
| **Exercise** | FTR | FTR | FTR | VWR | VWR | VWR | VWR |
| **Intervention time (weeks)** | 4  (5 days/wk) | 6  (5 days/wk) | 6  (5 days/wk) | 6  (5 days/wk) | 12  (7 days/wk) | 12  (7 days/wk) | 5  (7 days/wk) |
| **Distance/session** | 1000 m | 480 m | 600-700-800 m | 5 836±132 m/night | 17 390±6,890 (Counts) | 11 714.28 m | 11 538.06 m |
| **Time/session** | 60 min | 40 min | 40 min | n.a. | n.a. | n.a. | 680 min |
| **Speed** | 12-24 m/min | 8-12 m/min | 15-20 m/min | n.a. | n.a. | n.a. | 18.67 m/min |
| **Exercise intensity** | High | Low | Moderate | n.a. | n.a. | n.a. |  |
| **Group training** | Yes | No | No | No | No | No | No |
| **Sample origin** | cecal | cecal | fecal | cecal | fecal | fecal | fecal |
| **16S rRNA sequencing** | V2-4-8, V3-6, and V7-9 sequencing | V3 and V5 sequencing | V6-V8 sequencing | V3 and V5 sequencing | TRFLP and pyrosequencing | TRF and V4 sequencing | PhyloChip Arrays |
| **Relevant taxa**  **(vs. Sedentary mice)** | ↓ *Ruminoccocus gnavus*  ↑*Parabacteroides*  ↑*Desulfovibrio* | ↑ *R. gnavus*  ↑*Clostridium*  ↑*Butyrivibrio*  ↑ *Oscillospira* ↑*Coprococcus* | ↓ *Ruminoccocus*  ↓ *Lactobacillus*  ↓*Clostridiales*  ↓*Parabacteroides*  ↑*Bacteroides*  ↑*Lachnospiraceae* | ↑*Coprococcus*  ↑*Turicibacter* | ↑ *Allobaculum spp.*  ↑ *Ruminoccocus*  ↑ *Clostridiales*  ↑*Akkermansia* | Exercise increased the *Bacteroidetes*:*Firmicutes* ratio in a manner that was  proportional to the distance run. | ↓ *Ruminoccocus* |

Table 1. Comparison of our study and previous studies regarding gut microbiota in mice that have undergone endurance exercise.

*This study includes both male (n=5) and female (n=6) mice, the rest of the studies were conducted on males. n.a.: Data not available.
